# Organization and remodeling of olfactory subsystems across development in the brilliant-thighed poison frog *Allobates femoralis*

**DOI:** 10.1007/s00441-026-04103-3

**Published:** 2026-11-12

**Authors:** Anyelet Valencia-Aguilar, Thomas Hassenklöver, Eva Ringler, Ivan Manzini

**Affiliations:** 1 Department of Animal Physiology and Molecular Biomedicine, Institute of Animal Physiology, Justus-Liebig-Universität Giessen, Heinrich-Buff-Ring 38, Giessen, 35392 Germany; 2 Division of Behavioural Ecology, Institute of Ecology and Evolution, University of Bern, Bern, 3032 Switzerland

**Keywords:** Olfactory epithelium, Olfactory bulb, Immunohistochemistry, G-protein, Metamorphosis

## Abstract

Olfaction is important for many ecological and social behaviors, but the development, organization, and molecular features of the olfactory system remain poorly understood in most anuran amphibians. Understanding developmental changes is particularly important because, during metamorphosis, the olfactory system must adapt to the very different conditions encountered by aquatic larvae and mainly terrestrial adults. In the brilliant-thighed poison frog *Allobates femoralis*, chemical cues influence behavior during both aquatic and terrestrial life stages, but the organization of its olfactory system has not yet been described. Here, we examined the anatomy and molecular features of the olfactory system of *A. femoralis* during development. We identified a main olfactory epithelium and a vomeronasal organ, together with a distinct buccal-exposed olfactory epithelium that is present only in larvae. We also found an additional larval sensory epithelium that is in close connection to the main olfactory epithelium. The different sensory epithelia showed distinct anatomical features and projected to defined regions of the main and accessory olfactory bulb. Immunohistochemistry revealed Gαo-expressing receptor neurons in all these epithelia, together with Gαo-positive axonal projections to the main and accessory olfactory bulb. Our study provides the first detailed description of the developing olfactory system of *A. femoralis*. These findings provide a basis for future studies linking the organization and function of the olfactory system to the olfaction-guided behaviors of this species.

**Supplementary Information:**

The online version contains supplementary material available at https://doi.org/10.1007/s00441-026-04103-3.

## Introduction

Olfaction allows animals to detect chemical cues related to food, reproduction, environmental conditions, and potential threats, playing an important role in many ecological contexts and social interactions (Ache and Young 2005; Kaupp 2010; Manzini et al. 2022). Although the basic organization of the olfactory system in vertebrates is broadly conserved, its peripheral and central components have diversified considerably among vertebrate lineages (Hayden and Teeling 2014; Manzini et al. 2022). Anuran amphibians typically have two olfactory pathways. The main pathway consists of the main olfactory epithelium (OE) projecting to the main olfactory bulb (OB), whereas the accessory pathway consists of the vomeronasal organ (VNO) projecting to the accessory OB. In many species, especially during larval stages, additional peripheral olfactory epithelia have been described (Jungblut et al. 2017, 2021; Weiss et al. 2021a).

Odorants are detected by olfactory receptor neurons (ORNs) with different morphological and molecular properties. The two most common morphological types are ciliated and microvillous ORNs. These neurons express olfactory receptors belonging to different receptor families and use distinct G-protein-dependent signal-transduction pathways (Kaupp 2010; Oka and Korsching 2011; Francia and Lodovichi 2021; Manzini et al. 2022). In many vertebrates, ciliated ORNs are associated with Gαolf-mediated signaling, whereas microvillous ORNs are often associated with Gαo or related G-proteins (Manzini et al. 2022). The distribution of these cell types and G-proteins differs among the various sensory epithelia and can therefore help to distinguish different olfactory subsystems (Jungblut et al. 2021; Weiss et al. 2021a; Manzini et al. 2022).

Among anuran amphibians, the olfactory system of *Xenopus laevis* has been studied most extensively. Work on this species has described the peripheral olfactory organ and OB in considerable detail and has identified the main morphological types of ORNs and their molecular and physiological properties (Manzini and Schild 2010; Gascuel and Amano 2013; Weiss et al. 2021a). In larvae, the main OE contains ORNs expressing Gαolf, Gαo, and Gαi. Gαolf is mainly associated with ciliated ORNs, whereas Gαo and Gαi are associated with microvillous ORNs. These ORN populations project to different glomerular regions of the main OB (Gliem et al. 2013; Weiss et al. 2021a). The larval VNO contains microvillous receptor neurons that express Gαo and project to the accessory OB (Hagino-Yamagishi et al. 2004; Weiss et al. 2021a).

During metamorphosis, the olfactory system of *X. laevis* is extensively remodeled (Dittrich et al. 2016). Adults of this species possess sensory epithelia in the principal and middle nasal cavities and in the VNO. The principal cavity epithelium is primarily involved in detecting airborne odorants and contains ciliated Gαolf-expressing ORNs. The middle cavity epithelium, which is involved in the detection of waterborne odorants, contains both ciliated Gαolf-expressing and microvillous Gαo-expressing ORNs. The adult VNO is solely composed of microvillous Gαo-expressing receptor neurons (Mezler et al. 2001; Hagino-Yamagishi et al. 2004; Nakamuta et al. 2011; Weiss et al. 2021a).

The olfactory organs of other anurans differ in several respects from those of *X. laevis*. In addition to the main OE and the VNO, many anuran larvae possess an OE patch that is directly exposed to the buccal cavity. This so-called buccal-exposed OE (bOE) is particularly common in larvae that obtain food by scraping or browsing on submerged surfaces (Jungblut et al. 2017, 2021; Weiss et al. 2021a). In many adult anurans, the OE of the middle cavity found in *X. laevis* is absent. In contrast, several adult anurans have a separate epithelial patch adjacent to the main OE, i.e., the recessus olfactorius that is thought to contribute to the detection of waterborne odorants (Nowack et al. 2013; Jungblut et al. 2021; Weiss et al. 2021a).

Information on the morphology of ORNs and G-protein expression in most anuran species remains limited. Where comparable tissues and developmental stages have been examined, e.g., in *Rhinella arenarum*, *Boana pulchella*, and *Bufo japonicus*, the distribution of ciliated and microvillous receptor neurons and the expression of Gαolf, Gαo, and other G-proteins generally resemble the patterns described in *X. laevis* (Jungblut et al. 2009, 2012, 2021; Weiss et al. 2021a). A notable exception occurs in the adult VNO of *B. japonicus*, where Gαolf-expressing receptor neurons are located apically, whereas Gαo-expressing receptor neurons occupy the middle-to-basal layers of the vomeronasal epithelium (Hagino-Yamagishi and Nakazawa 2011). Both ciliated and microvillous ORNs have also been described in the larval bOE and in the recessus olfactorius of adult anurans (Weiss et al. 2021a). However, G-protein expression in these two epithelial regions has not yet been resolved.

Functional studies in *X. laevis* provide an important context for understanding the importance of the molecular organization of the olfactory system. They have established links between distinct receptor neuron populations and their associated transduction machinery in olfactory processing. These studies span from individual receptor neurons through the OB to higher olfactory centers, employing electrophysiological and calcium-imaging techniques (e.g., Czesnik et al. 2001; Manzini et al. 2002; Manzini and Schild 2003; Offner et al. 2020, 2023; Weiss et al. 2021b). In this species, distinct populations of ciliated and microvillous receptor neurons are associated with different molecular components of odor transduction and project to anatomically segregated regions of the OB, providing two partially distinct pathways for olfactory processing (Manzini and Schild 2010; Gliem et al. 2013). These pathways are further modified during metamorphosis, when the larval water nose undergoes extensive remodeling, and the principal cavity becomes specialized for aerial olfaction (Weiss et al. 2021a, 2021b), thus providing a framework for interpreting the functional significance of G-protein expression in other olfactory epithelia.

Behavioral studies complement these cellular and molecular findings by linking them to olfactory function. In *X. laevis*, research has focused mainly on chemotaxis toward waterborne amino acids, showing that olfactory attraction is already evident in larvae and persists throughout metamorphosis (Hawkins et al. 2017; Terni et al. 2017; Weiss et al. 2021b). Although these studies demonstrate functional olfactory behavior throughout development, they provide limited insight into olfaction’s contribution to more complex behavioral decisions. Studies in other anurans, particularly poison frogs and tree frogs, indicate that olfactory cues can contribute to a broader repertoire of ecologically relevant behaviors including reproductive and social communication, predator avoidance, resource localization, and parent–offspring interactions (Caldwell and Araujo 1998, Schulte et al. 2011, 2015, Schulte and Rössler 2013, Starnberger et al. 2013, Schulze et al. 2024, Surber-Cunningham et al. 2024). Yet the neural mechanisms underlying these behaviors remain largely unresolved, as the anatomical, molecular, and functional organization of the olfactory system remains poorly characterized in many of these species.

The brilliant-thighed poison frog *Allobates femoralis* illustrates particularly well this imbalance between behavioral evidence for olfactory function and our limited knowledge of the underlying anatomical, molecular, and functional organization of the olfactory system. It is a small, diurnal species with territorial behavior and complex parental care, including the transport of larvae to aquatic deposition sites (Ringler et al. 2009, 2012). Larvae of *A. femoralis* use visual and chemical cues to detect and avoid predators, whereas adult males use olfactory information to locate water bodies suitable for deposition of larvae (Szabo et al. 2021; Serrano-Rojas and Pašukonis 2021; Peignier et al. 2024). Adult females also appear to use chemical cues to locate feeding sites (Ringler, unpublished observation). Despite the importance of olfaction throughout the life cycle of *A. femoralis*, its olfactory system has not previously been described.

In the present study, we examined the organization of the olfactory system of *A. femoralis* during larval development. We characterized the anatomy and cellular composition of the different sensory epithelia, traced their projections to the main and accessory OB, and analyzed the distribution of Gαo-expressing receptor neurons using immunohistochemistry. In particular, we asked whether the organization and Gαo expression patterns found in *A. femoralis* resemble those described in other anurans or represent species-specific features. The data presented in this manuscript provide the first detailed anatomical description of the olfactory system of *A. femoralis*. It, therefore, establishes a basis for future studies linking the organization and function of the olfactory system to the olfaction-guided behaviors of this species.

## Materials and methods

### Animal and tissue preparation

*Allobates femoralis* larvae used in this study were obtained from the breeding colony at the Ethological Research Station of the Institute of Ecology and Evolution at the University of Bern (Switzerland). Developmental stages were determined according to Gosner (1960). Animals were grouped into three developmental stages: premetamorphotic animals (stages 25–30, absent or rudimentary limb buds), prometamorphotic animals (stages 31–41, no externally visible forelimbs), and animals at the metamorphic climax (stages 42–46, characterized by tail resorption). Before experimental procedures, the animals were anesthetized using 0.02% MS-222 (ethyl 3-aminobenzoate methanesulfonate, TCI Germany) in tap water. For tissue preparation, anesthetized animals were killed by severing the transition between the brainstem and spinal cord. All animal procedures were performed following the guidelines for laboratory animal research of the Institutional Care and Use Committee of the University of Giessen (882_M).

### Labeling of olfactory receptor neurons

Larvae were anesthetized and euthanized as described above. The olfactory system was dissected, and the olfactory nerve was transected. Biocytin crystals (ε-biotinoyl-L-lysine, Molecular Probes, Thermo Fisher Scientific, B1592) were placed into the transected olfactory nerve, which was then covered with tissue adhesive (Histoacryl L, Braun, 1,050,060). The tissue was then placed in frog Ringer solution (98 mM NaCl, 2 mM KCl, 1 mM CaCl_2_, 2 mM MgCl_2_, 5 mM Na-pyruvate, 5 mM glucose, 10 mM HEPES, pH 7.8) for 2 h. After this labeling procedure, a block of tissue containing the olfactory organ, the olfactory nerves, and the anterior telencephalon was cut out and fixed in 4% formaldehyde for 2 h at room temperature. The tissue block was then washed three times, each for 10 min in 150 µl PBS, embedded in 5% low-melting-point agarose (Sigma-Aldrich), and sliced horizontally into 150 µm slices using a vibratome (VT 1200 S; Leica, Bensheim, Germany). The obtained slices were washed three times, each for 10 min in 150 µl PBS-T to permeabilize the tissue, followed by a 1-h incubation in 150 µl PBS-T with 2% normal goat serum (NGS, MP Biomedicals) to block nonspecific binding sites. For tissue clearing, the slices were bleached by incubation for 60 min at 30 °C in 2% H_2_O_2_ diluted in 1 × Tris–HCl (pH 8, see Chung et al. 2016). The bleaching solution was washed off five times, each for 5 min in 150 µl PBS, and incubated in Alexa Fluor 488 conjugated with streptavidin (Life Technologies, S11223, 1:400 in PBS-T) for 5 h.

### Immunohistochemistry

Tissue slices were prepared as above, permeabilized, and nonspecific binding sites were blocked as described above. The slices were then incubated with one of the following antibodies (1:100): Calretinin, a marker for ORNs (Swant, rabbit polyclonal, CR7697), or Gαo (Santa Cruz Biotech, mouse monoclonal A-2, sc-13532) in 150 µl PBS-T 2% NGS at 4 °C for 72 h. After the incubation, the slices were washed 5 times in 150 µl PBS. To remove tissue pigments, the slices were cleared as described above. The tissue was then incubated with the following secondary antibodies (1:250): Alexa Fluor 488 goat anti-mouse (Invitrogen, A11001), Alexa Fluor 488 goat anti-rabbit (Invitrogen, A11008), Alexa Fluor 594 goat anti-mouse (Invitrogen, A11005), or Alexa Fluor 594 goat anti-rabbit (Invitrogen, A11012) in PBS with 2% NGS at 4 °C for 72 h. Then, the secondary antibodies were washed off five times, each for 5 min in 150 µl PBS. Samples with a single primary label were incubated with 10 μg/ml propidium iodide (Invitrogen, P3566) for 15 min to label cell nuclei. Subsequently, these samples were washed three times with PBS for 10 min each. Following this, slices were mounted on slides using mounting medium (Dako, S3023, Hamburg, Germany). Negative control experiments were performed for each primary antibody by omitting the respective antibody, and no staining was detected in any of these controls.

### Image acquisition and processing

Images were acquired as virtual image stacks with multiple color detection channels and a z-resolution of 1–2 μm using multiphoton microscopy (Nikon A1R-MP) at an excitation wavelength of 780 nm. Adjustments to brightness and contrast were performed in ImageJ (v 1.54f). Where needed, multiple image stacks were combined using a stitching algorithm (Preibisch et al. 2009). Images in figures are presented as maximum-intensity projections of the z-stacks along the *z*-axis generated in ImageJ.

## Results

### Anatomical characterization of the olfactory system of *Allobates femoralis*

To characterize the anatomical organization of the olfactory system in *A. femoralis*, we primarily used calretinin immunohistochemistry. In larval and adult *X. laevis*, calretinin is expressed in a large population of receptor neurons in the main OE and VNO, including their axonal projections to the OB (Kahl et al. 2024). We also observed widespread calretinin labeling in the *A. femoralis* olfactory system, and this allowed us to determine the anatomical location of the peripheral sensory epithelia and to characterize the projections of their receptor neurons to the OB.

#### Premetamorphosis (stages 25–30)

**Fig. 1:**
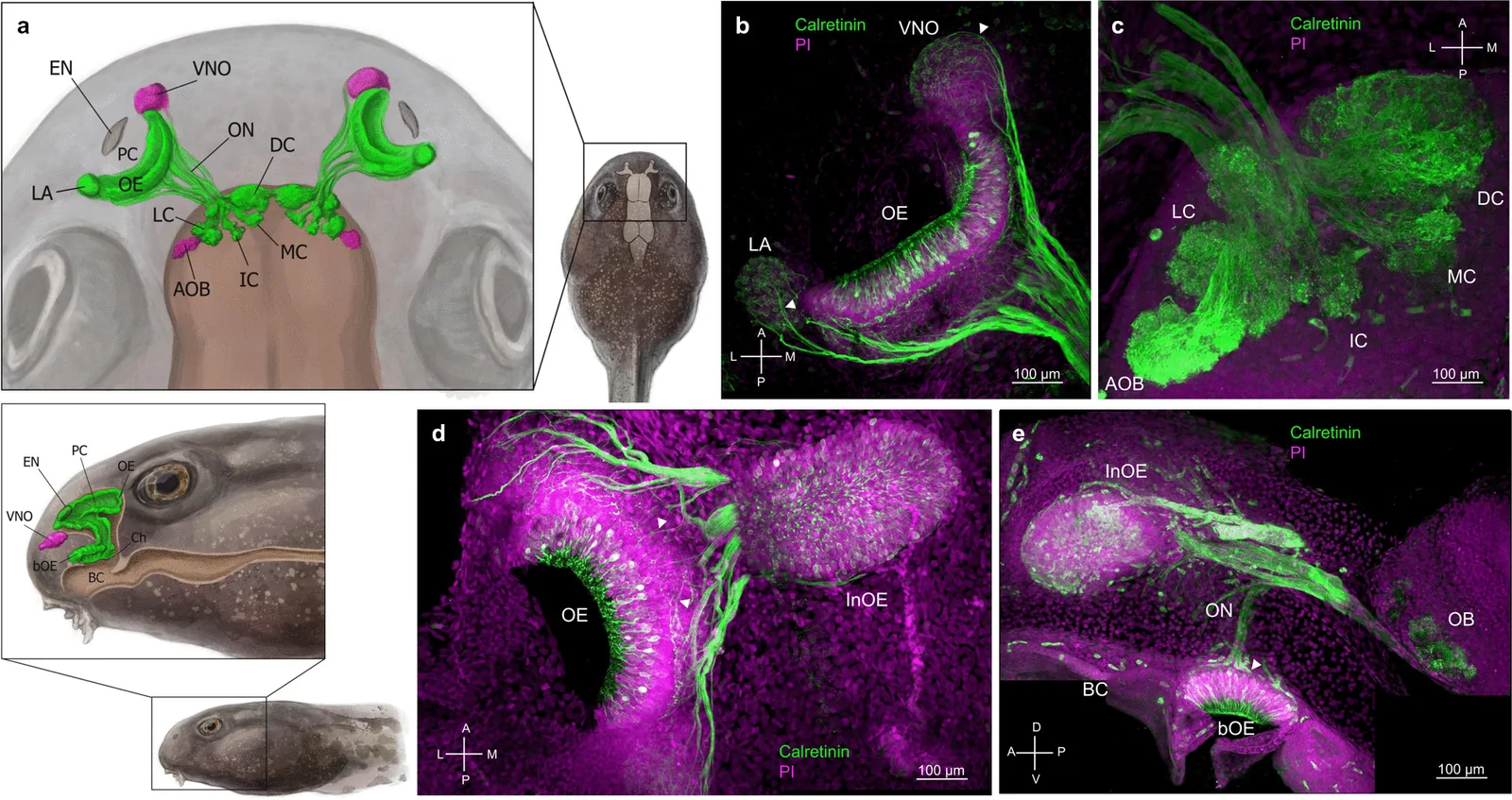
Anatomical organization of the olfactory organ of premetamorphic *Allobates femoralis* (stages 25–30). Immunohistochemical staining for calretinin (green) labels ORNs throughout the olfactory organ, while nuclei are counterstained with propidium iodide (purple). **a** Dorsal (above) and lateral (below) views of a larva with a scheme of the olfactory system. The OE and bOE are included in the same cavity of the olfactory system, but the connection of these two structures was not resolved. **b** Olfactory organ showing the main OE, vomeronasal organ (VNO), and lateral appendage (LA). **c** Glomerular clusters in the main OB, including the dorsal cluster (DC), medial cluster (MC), intermediate cluster (IC), lateral cluster (LC), and the accessory OB. **d** Horizontal view of the main OE showing the funnel-like invagination (InOE) that extends caudally from the cranial portion of the main OE. **e** Sagittal section of the olfactory system shows the caudal part of the main OE (InOE) and the buccal-exposed olfactory epithelium (bOE) in the buccal cavity. White arrowheads in **b**, **d**, **e** indicate axons of ORNs. A, anterior; P, posterior; L lateral, M, medial; D, dorsal; V, ventral; BC, buccal cavity; EN, external nares; Ch, choana; PC, principal cavity; ON, olfactory nerve. Illustrations: Javier Lazaro

In early stages of *A. femoralis*, the small external nares are located dorsolaterally at the rostral end of the nasal cavity and are oriented anterolaterally. The olfactory system comprises the olfactory organ, located adjacent to the nares, and the olfactory bulb (OB), situated in the anterior region of the brain. The olfactory organ is connected to the OB by the olfactory nerves (Fig. 1a). The nasal cavity contains three distinct sensory epithelia, including the main OE, the VNO situated anteromedially to the main OE, and an additional sensory epithelium contained within a spherical structure lateral to the main OE, which we termed lateral appendage (LA). These sensory structures are located within a semicircular principal cavity (Fig. 1a, b).

The main OE comprises two anatomically distinguishable regions: a cranial portion and a caudally extending, characteristic funnel-shaped invagination (Fig. 1d). The VNO is visible as a spherical structure located in an anteroventral region of the nasal cavity, in close vicinity to the cranial portion of the main OE (Fig. 1b). The epithelium of the LA, on the other hand, is located within a small, oval-shaped structure situated posterolateral to the main OE (Fig. 1b). In addition, we found a bOE within the buccal cavity (Fig. 1e) that forms a discrete patch of sensory epithelium along the dorsal wall of the buccal cavity (Fig. 1e).

Axons from ORNs in the main OE, the bOE, and the LA converge to form the olfactory nerve and project to the main OB (arrowheads Fig. 1b, d, e). Thereby, axons of ORNs of the bOE project dorsally from the buccal cavity and join the olfactory nerve approximately halfway between the main OE and the OB (Fig. 1e). Within the main OB, receptor neuron axons terminate into four characteristic glomerular clusters, i.e., the lateral cluster (LC), intermediate cluster (IC), medial cluster (MC), and dorsal cluster (DC, Fig. 1c). At stage 25, following hatching, the dorsal clusters of both hemispheres are separated (Fig. S1). Axons of receptor neurons of the VNO (Fig. 1b) join the olfactory nerve and terminate in the accessory OB that is located laterally to the main OB (Fig. 1c).

#### Prometamorphosis (stages 31–41)

**Fig. 2:**
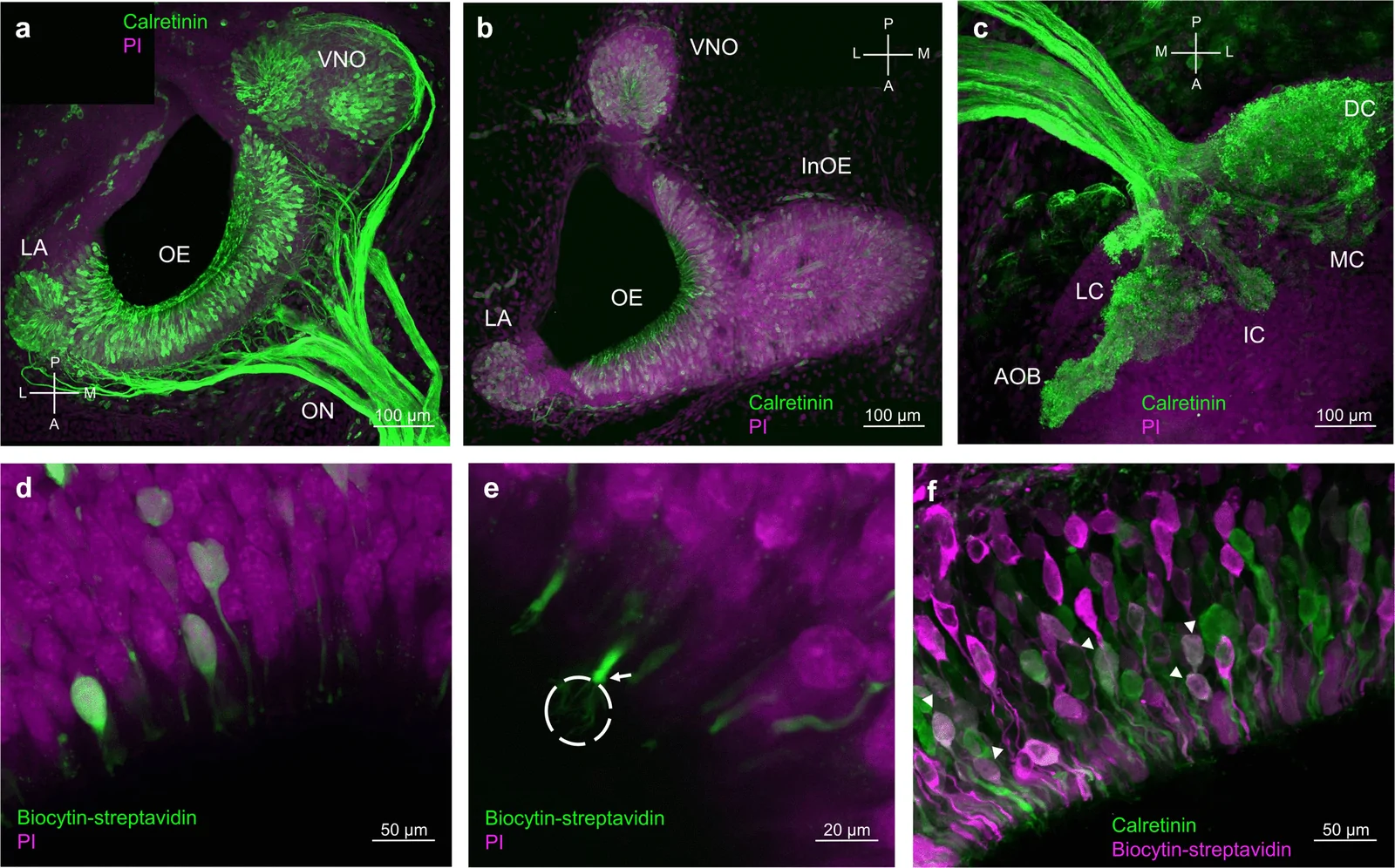
Anatomical organization of the olfactory organ of prometamorphic *Allobates femoralis* (31–41 stages). **a** Image showing strong calretinin-like signals (green) in the main OE, vomeronasal organ (VNO), lateral appendage (LA), and the initial segment of the olfactory nerve (ON). **b** VNO, LA, and cranial and caudal regions of the main OE. **c** Glomerular clusters in the main OB, including the dorsal cluster (DC), medial cluster (MC), intermediate cluster (IC), lateral cluster (LC), and accessory OB. **d** Olfactory receptor neurons (ORNs) of the main OE labeled with biocytin-streptavidin staining (green) and cell nuclei stained with propidium iodide (PI, purple). **e** Olfactory receptor neuron appendages (white dotted circle) and dendritic knobs (white arrow). **f** Double labeling of ORNs of the main OE with calretinin (green) and biocytin-streptavidin (purple). Arrowheads indicate neurons positive for both markers. A, anterior; P, posterior; L, lateral; M, medial

As observed in earlier developmental stages, the external nares remained dorsolaterally positioned and anterolaterally oriented, while both the principal and buccal cavities slightly increased in size. The principal cavity became more elliptical in shape (compare Figs. 1b and 2a). At developmental stage 33, examination across different focal planes revealed a structure surrounding the medial region of the principal cavity that was consistent with a cartilaginous capsule (not shown). Vomeronasal glands were also evident within the connective tissue overlying the VNO (not shown). The LA showed no noticeable positional change and remained visible as an oval-shaped structure, as in earlier developmental stages (Fig. 2b). At stage 41, the bOE remained visible along the dorsal surface of the buccal cavity but was visually reduced in extent (not shown). The organization of the cranial region of the main OE remained similar to earlier stages, and the caudal region of the main OE remained deeply invaginated and retained its funnel-like shape (Fig. 2b). The gross organization of both the glomerular clusters within the main OB and the glomeruli in the accessory OB remained unchanged compared with earlier stages (Fig. 2c).

Olfactory receptor neurons in the main OE, labeled via an olfactory nerve backfill (biocytin-streptavidin staining), exhibited a bipolar morphology, with a cell body and an apical dendrite terminating in a characteristic knob-like structure bearing apical appendages (Fig. 2d, e). Calretinin labeling and biocytin backfills clearly revealed the morphology of ORNs in the main OE, including their cell bodies, dendrites, and apical knobs (Fig. 2f). Only a subpopulation of ORNs was double-labeled by both methods, indicating that calretinin does not label all ORNs (Fig. 2f).

#### Metamorphic climax (stages 42–46)

**Fig. 3:**
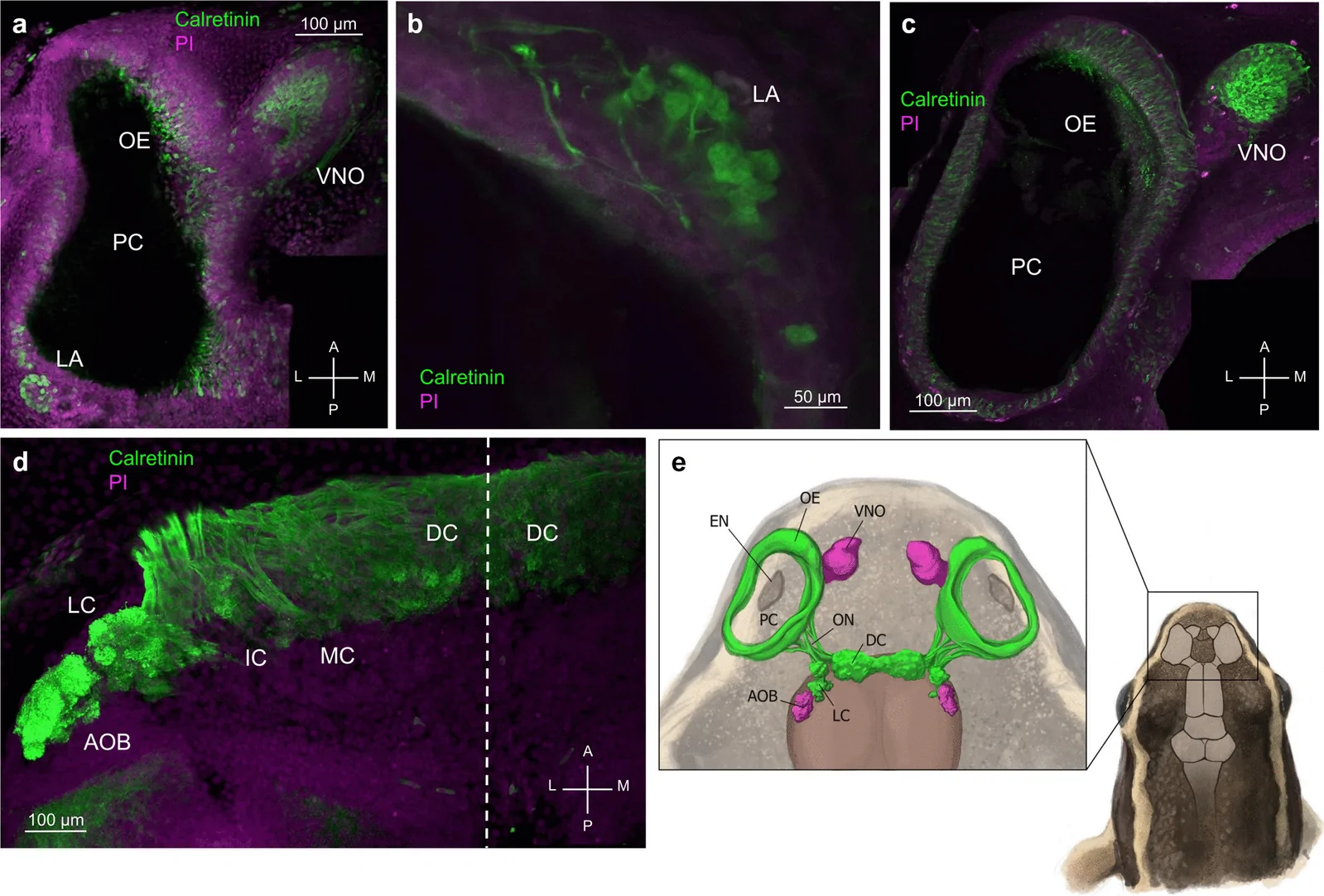
Anatomical organization of the olfactory organ in metamorphic *Allobates femoralis* (42–46 stages). Immunohistochemical staining for calretinin (green) shows ORNs throughout the olfactory organ, while cell nuclei are stained with propidium iodide (purple). **a** Appearance of the main OE, vomeronasal organ (VNO), and lateral appendage (LA) at developmental stages 42–43. **b** Higher-magnification view of the rather disorganized LA at stages 42–43. **c** Horizontal view of the main OE at developmental stages 45–46. **d** Glomerular clustering in the main OB, including the dorsal cluster (DC), medial cluster (MC), intermediate cluster (IC), lateral cluster (LC), and accessory OB. Note that the IC and MC are no longer clearly identifiable and that the DCs are fused. The dashed line marks the interhemispheric midline. **e** Horizontal view of a metamorphic individual is shown with a schematic illustration highlighting the nasal region. A, anterior; P, posterior; L, lateral; M, medial. Illustration: Javier Lazaro

As metamorphosis progresses, through stages 42 and 43, the principal cavity underwent significant morphological changes, transforming from a half-moon shape to an elongated, oval structure (compare Figs. 1b and 3a). From stage 44 onward, the principal cavity further enlarged and assumed a cylindrical shape (Fig. 3a). After metamorphosis, the funnel-like invagination of the main OE (caudal part of the main OE, Fig. 3c) and the bOE were no longer visible. The LA progressively lost its organization and gradually disappeared (see Fig. 3b, c). The VNO, on the other hand, remained located in an anteroventral position relative to the principal cavity (Fig. 3c).

At stage 46 (Fig. 3c, e), the overall morphology of the olfactory organ was already similar to that observed in adult animals. In juveniles, the main OE formed a continuous epithelial layer lining the principal cavity and appeared particularly prominent along its medial and lateral margins (Fig. 3c). The VNO remained located anteroventral to the principal cavity and displayed a rounded morphology with a dense population of receptor neurons (Fig. 3c). The cavity of the VNO was visually connected to the principal cavity through a duct-like structure (not shown).

The OB also underwent notable changes during the later stages of metamorphosis, particularly in the distribution and appearance of its glomerular clusters. The DCs of the left and right hemispheres fused to form a single prominent dorsal cluster, whereas the ICs and MCs became progressively less distinct and increasingly difficult to resolve during stages 43–46 (Fig. 3d). Their complete disappearance, however, could not be conclusively established. In contrast, the lateral cluster and the glomeruli of the accessory OB remained prominent throughout development, including at the latest stages examined.

### Expression of Gαo in the olfactory system of *Allobates femoralis*

**Fig. 4:**
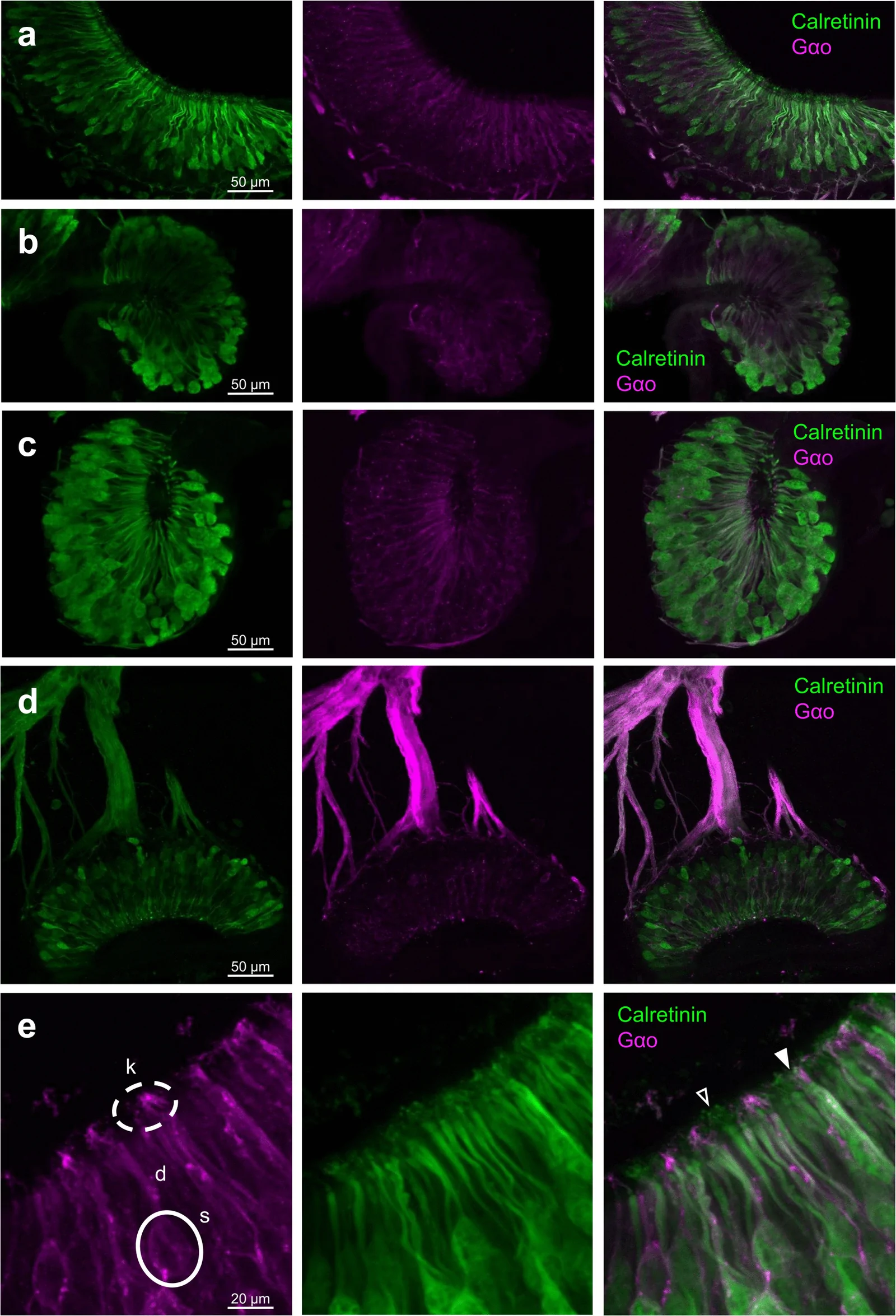
G*αo*-positive olfactory receptor neurons in the sensory epithelia of premetamorphic *Allobates femoralis* (25–30 stages). Gα*o-*positive ORNs (purple) were found throughout the main OE (**a**), lateral appendage (LA, **b**), vomeronasal organ (VNO, **c**), and buccal-exposed olfactory epithelium (bOE, **d**). In **d**, axons leaving the buccal-exposed epithelium (bOE) were clearly Gαo-positive. In all epithelia, Gα*o* immunoreactivity was primarily associated with dendritic and axonal processes of receptor neurons (see also Fig. 6). **e** Higher-magnification views of the main OE illustrating the differential subcellular localization of both markers. Double immunolabeling with antibodies against calretinin (green) and Gα*o* demonstrated distinct, partially overlapping expression patterns in receptor neurons. Calretinin labeled the whole receptor neurons, whereas Gα*o* immunoreactivity was mostly present in olfactory knobs and their appendages (dotted circle). Open arrowheads indicate Gα*o*-negative olfactory knobs, whereas closed arrowheads indicate Gα*o*-positive olfactory knobs. S, soma (white circle); k, knob; d, dendrite

The antibody against Gαo labeled receptor neurons within the olfactory epithelia, as well as their axons. Gαo expression was detected in all olfactory epithelia throughout development of *A. femoralis*, i.e., in the main OE (Figs. 4a and 6), LA (Fig. 4b), VNO (Figs. 4c and 6), and bOE (Fig. 4d). To better determine which cellular compartments of the receptor neurons were Gαo-positive, we performed double immunostaining for Gαo and calretinin. While calretinin labeled multiple cellular compartments in most receptor neurons across all sensory epithelia, Gαo immunoreactivity showed a more restricted, patchy distribution and was limited to a subpopulation of receptor neurons. Pronounced Gαo immunoreactivity was observed in the apical olfactory knobs of a subpopulation of receptor neurons and their appendages but was also present in dendrites, cell bodies, and axons (Fig. 4a, e). This pattern is particularly evident in Fig. 4e, which shows a higher-magnification view of a region of the main OE. The higher magnification provides a clearer view of Gαo-positive olfactory knobs and their apical appendages. Similar results were also obtained in the sensory epithelia of prometamorphic and metamorphic stages (see Fig. 6).

**Fig. 5:**
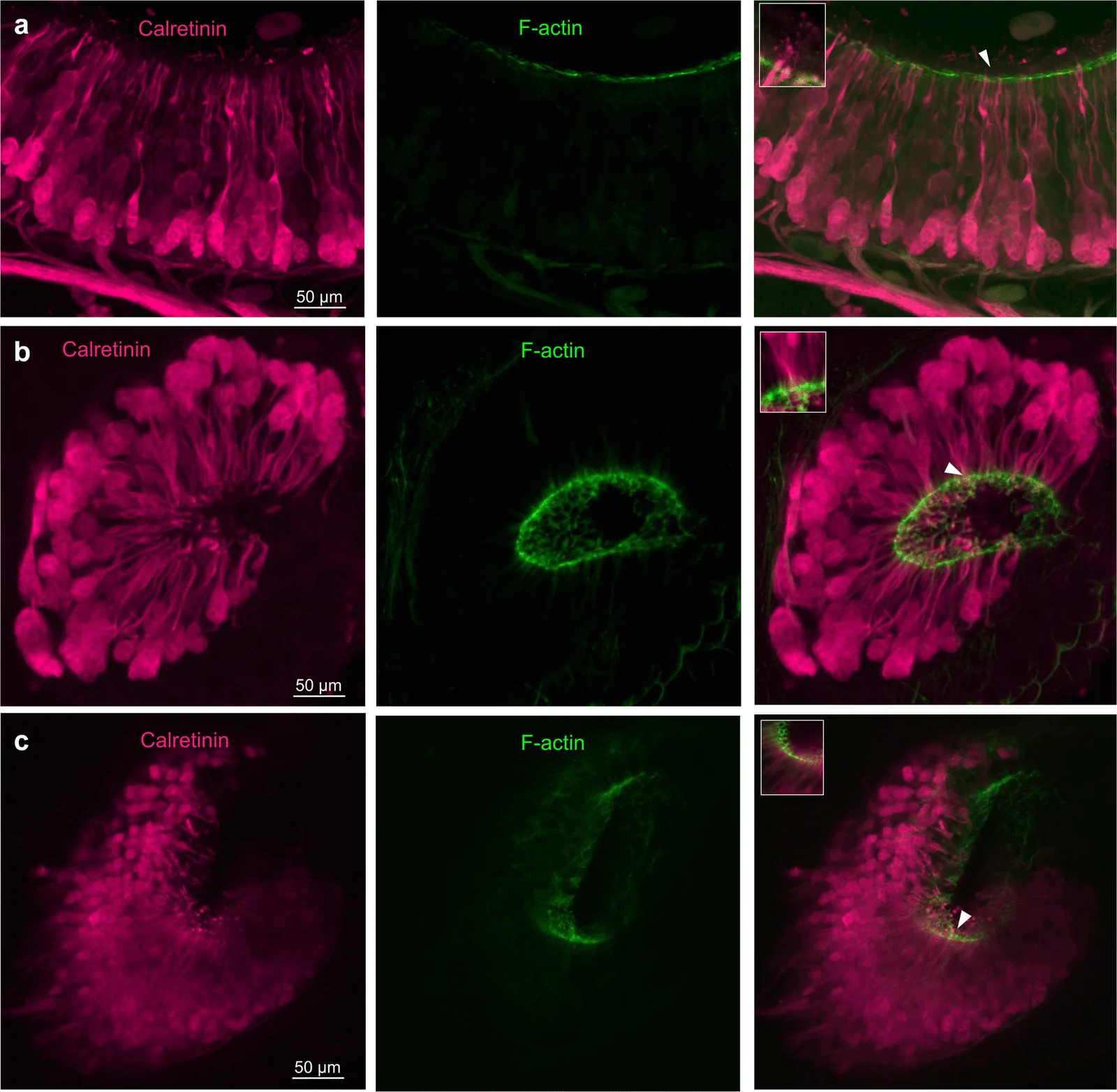
F-actin distribution in olfactory epithelia of *Allobates femoralis* of different developmental stages. Calretinin-positive ORNs (purple) and F-actin-rich cellular appendages (green) in the main OE (**a**, premetamorphic stage), lateral appendage (LA, **b**; prometamorphic stage), and vomeronasal organ (VNO, **c**; metamorphic stage). Arrowheads indicate calretinin-positive ORNs associated with F-actin-rich apical appendages. The regions indicated by the arrowheads are shown at higher magnification in the insets in the right-hand side images

Because Gαo immunoreactivity was also detected in olfactory knobs and their apical appendages, we further examined the morphology of these appendages to determine which morphological types of receptor neurons are present in the different sensory epithelia of *A. femoralis*. In sections of the different epithelia, double labeling with calretinin and fluorescently conjugated phalloidin, which labels F-actin in microvilli, revealed abundant F-actin-positive olfactory knobs. Cilia-like structures were visible in the main OE (Fig. 5a, premetamorphic stage), whereas the LA (Fig. 5b, prometamorphic stage) and VNO (Fig. 5c, metamorphic stage) showed prominent F-actin-positive microvilli but no clearly identifiable cilia. This suggests that these epithelia predominantly or exclusively contain microvillous receptor neurons. We did not observe changes in this pattern over development.

**Fig. 6:**
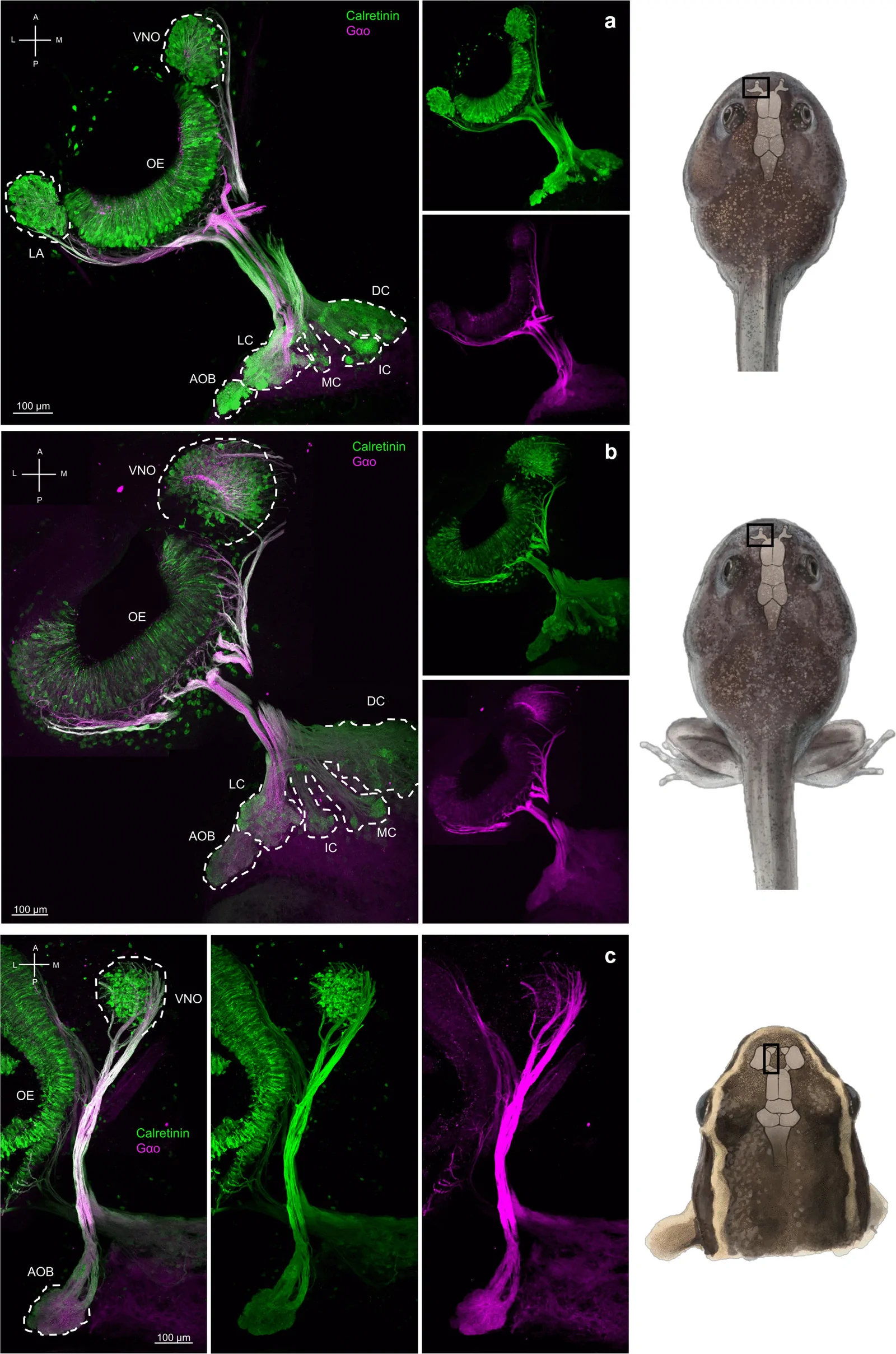
Gα*o* expression in the olfactory system of *Allobates femoralis* across developmental stages. Representative images from premetamorphic (**a**), prometamorphic (**b**), and metamorphic (**c**) stages showing main and accessory systems. Gα*o* immunoreactivity is shown in purple, calretinin immunoreactivity is shown in green. In the premetamorphic and prometamorphic stages, Gαo-positive receptor neuron axons are present in all sensory epithelia and innervated the accessory OB and the lateral cluster (LC) of the main OB. Only weak labeling was found in the other glomerular clusters. Note that in (**b**) the lateral appendage (LA) is not in the field of view. In metamorphic animals, Gα*o* signal was mainly located in the vomeronasal organ (VNO) and to some extent also in the main OE. Gα*o*-positive axons clearly innervated the accessory OB and to a lesser extent the dorsal cluster (DC). Dashed outlines delimit individual glomerular clusters in the OB. A, anterior; P, posterior; L, lateral; M, medial. Illustrations of the animals: Javier Lazaro

**Fig. 7:**
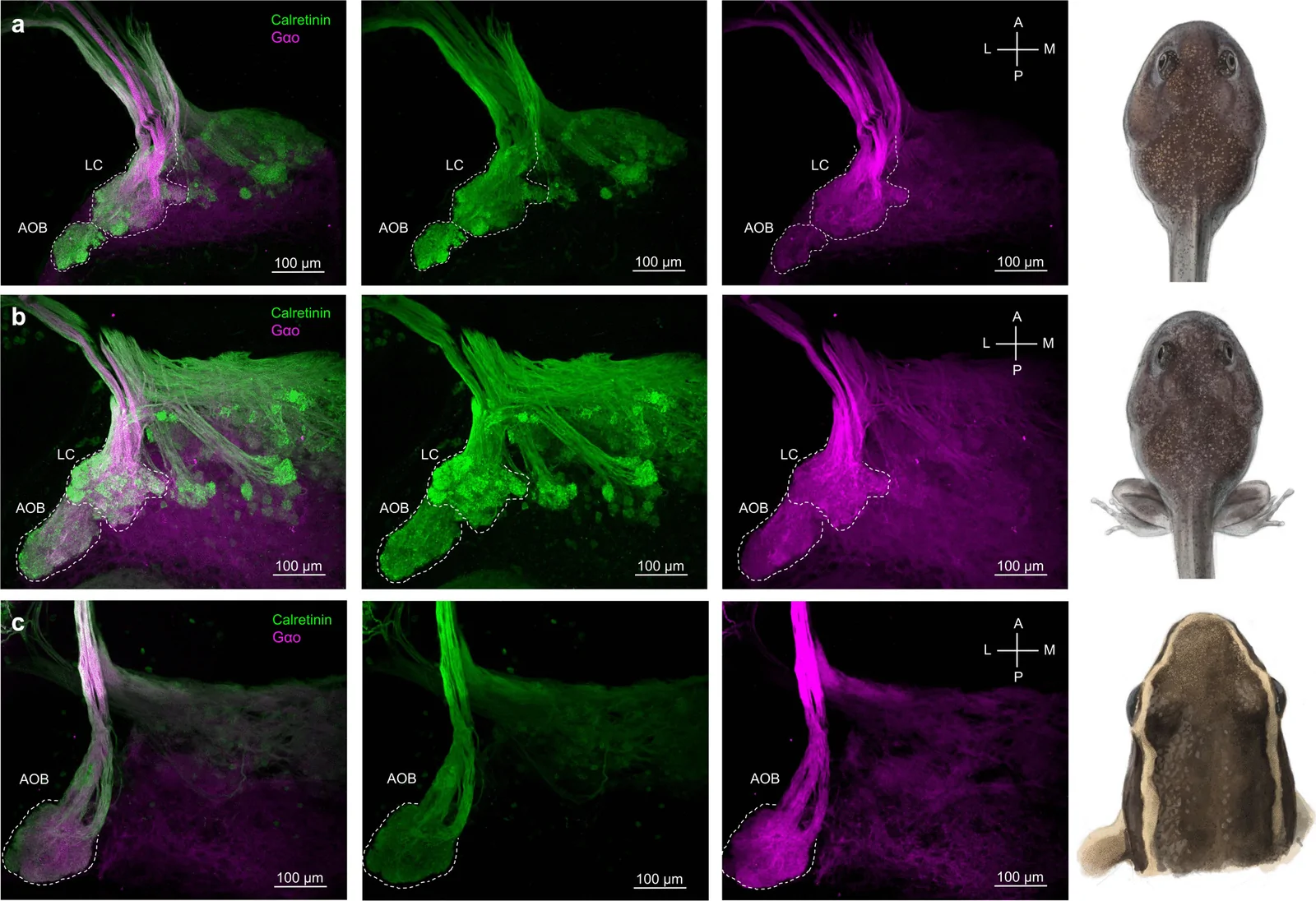
Innervation of the main and accessory olfactory bulb by Gα*o*-positive axons of receptor neurons of *Allobates femoralis* across developmental stages. Representative images from premetamorphic (**a**), prometamorphic (**b**), and metamorphic (**c**) stages showing the main OB and accessory OB. G*αo* immunoreactivity is shown in purple, calretinin immunoreactivity is shown in green. In the premetamorphic and prometamorphic stages, Gαo-positive receptor neuron axons innervated the accessory OB and the lateral cluster (LC) of the main OB. No evident labeling was found in the other glomerular clusters. In metamorphic animals, Gα*o*-positive axons innervated the accessory OB. Weak labeling was also found in other regions of the OB. Dashed outlines delimit individual glomerular clusters in the OB. A, anterior; P, posterior; L, lateral; M, medial. Illustrations of the animals: Javier Lazaro

To determine the connectivity of the olfactory organ to the OB, we performed double labeling for calretinin and Gαo in animals at premetamorphic, prometamorphic, and metamorphic stages (Fig. 6). In premetamorphic stages, Gαo-expressing receptor neurons were present in the main OE, LA, VNO, and bOE (Fig. 6a). We observed strong Gαo-positive axons terminating in the LC and the accessory OB (Fig. 7a). The remaining glomerular clusters of the main OB, i.e., the DC, MC, and IC, were largely Gαo-negative, although we cannot exclude that some Gαo-positive fibers terminated in these regions (Fig. 7a). Almost identical results were obtained in prometamorphic stages (Figs. 6b and 7b). During later developmental stages (stages 42–46), Gαo-positive axons still strongly innervated the accessory OB, but a clear innervation of the LC was no longer apparent (Figs. 6c and 7c). Instead, some Gαo-positive axons projected to the DC (Figs. 6c and 7c).

## Discussion

Our observations in *A. femoralis* are consistent with the established view that anuran metamorphosis involves remodeling of the olfactory periphery and its projections to the olfactory bulb. During development, the main OE and VNO remain recognizable through the larval-to-juvenile transition, whereas the bOE and LA are no longer evident in juveniles. The OB also undergoes changes in the organization and delineation of individual glomerular clusters. Alongside these anatomical changes, ciliated and microvillous ORNs remain recognizable, and G*αo* immunoreactivity remains detectable in these neurons throughout development.

### Organization of the larval peripheral olfactory epithelia

The main OE constitutes the principal sensory surface of the larval olfactory organ in anurans and lines the PC, where it is exposed to the surrounding aquatic environment (Jungblut et al. 2017, 2021; Quinzio and Reiss 2017; Weiss et al. 2021a). Although this organization is conserved among anuran larvae, the extent and spatial arrangement of the main OE within the PC vary among species (Jermakowicz et al. 2004; Jungblut et al. 2009, 2011, 2017, 2021; Benzekri and Reiss 2012; Quinzio and Reiss 2017). In *X. laevis*, *Ascaphus truei*, and *Ceratophrys cranwelli*, the main OE forms a relatively continuous sensory surface along the PC, with *C. cranwelli* showing continuity specifically along the dorsomedial PC edge (Dittrich et al. 2016; Benzekri and Reiss 2012; Quinzio and Reiss 2017). In contrast, the main OE is divided into dorsal and ventral portions in some bufonid and ceratophryid species (Jermakowicz et al. 2004; Jungblut et al. 2011; Quinzio and Reiss 2017). In *A. femoralis*, the main OE also forms a continuous epithelial surface within the PC but extends caudally into a prominent funnel-shaped region with distinguishable cranial and caudal portions. The funnel-shaped region is continuous with the main OE and therefore represents a caudal extension of this epithelium rather than a separate sensory compartment.

Posterior-lateral to the main OE, *A. femoralis* has an additional sensory epithelial region, the LA, which remains distinguishable throughout larval development. A similarly positioned structure, termed the lateral appendix, has been described as a larval component of the olfactory organ in *Rana nigromaculata*, *Alytes obstetricans*, and several ceratophryid species (Tsui 1946; Yvroud 1966; Quinzio and Reiss 2017). In these species, the lateral appendix forms a lateral or dorsolateral epithelial outgrowth associated with the principal olfactory cavity, although its sensory properties and central projections remain poorly characterized. The comparable position of the LA relative to the main OE and PC in *A. femoralis* therefore raises the possibility that these structures are anatomically related. Whether these structures are homologous remains to be determined through comparative anatomical and molecular analyses. Functionally, however, the role of the LA remains unclear, including which odorants its ORNs detect and how they contribute to olfactory processing.

Axons from the LA projecting toward the OB suggest that in *A. femoralis* provides an additional source of olfactory input to the brain, raising the possibility that it conveys information distinct from, or complementary to, that provided by the main OE. The nature of this additional input is especially important to consider because olfactory-associated sensory systems can incorporate modalities beyond chemosensation. Receptor neurons in the mouse Grüneberg ganglion, for example, respond to chemical and thermal stimuli (Brechbühl et al. 2008, 2020; Mamasuew et al. 2008), while in *X. laevis* tadpoles, thermal and mechanical stimulation can activate olfactory-bulb circuits receiving input from the olfactory epithelium (Kludt et al. 2015; Brinkmann and Schild 2016; Brinkmann et al. 2016). These studies do not provide evidence that the LA in *A. femoralis* is similarly multimodal, but they broaden the potential functional roles of an additional olfactory pathway. This possibility may be especially relevant given the ecology of *A. femoralis* larvae, which develop in temporary and structurally heterogeneous aquatic microhabitats where local environmental conditions can vary substantially (Ringler et al. 2013; McKeon and Summers 2013). Determining how the LA contributes to sensory processing in these variable environments will therefore be an important next step.

The larval olfactory organ of *A. femoralis* also includes the bOE, which is exposed to the buccal cavity. The bOE has been described in several anuran larvae and is particularly developed in species with grazing or scraping feeding modes, suggesting a role in the detection of chemical cues associated with the feeding environment (Jungblut et al. 2011, 2017, 2021). In *A. femoralis*, the bOE contains ORNs whose axons join the olfactory nerve, demonstrating a direct connection with the olfactory pathway. Its exposure to the buccal cavity, together with its sensory connection to the olfactory pathway, supports the hypothesis that the bOE contributes to chemical assessment of the feeding environment. This proposed role raises the question of which chemical cues are detected by bOE receptor neurons and whether their responses are specifically associated with feeding or encompass a broader range of environmental stimuli.

In contrast to these larval specializations, the VNO is a conserved element of the anuran olfactory system that develops during late embryonic stages and forms a distinct sensory epithelium within the olfactory organ (Jungblut et al. 2012, 2021; Weiss et al. 2021a). In *A. femoralis*, the VNO remains anatomically distinct from the main OE throughout larval development, and its receptor neurons project to the AOB, as described in other anuran larvae (Jungblut et al. 2012, 2021; Weiss et al. 2021a). The functional role of the VNO during the larval period, however, remains unclear. Traditionally, the main OE and VNO have been associated with different classes of chemical cues, but experimental evidence indicates that their sensory responses can overlap. In larval *X. laevis*, for example, sulfated steroids are detected by both the main OE and VNO, demonstrating that these two olfactory subsystems can respond to common chemical cues (Sansone et al. 2014). Thus, anatomical separation does not necessarily imply strict functional segregation, and whether the VNO makes a distinct sensory contribution during development in *A. femoralis* remains to be determined.

### Remodeling of the olfactory system during metamorphosis

Metamorphosis reorganizes the larval olfactory epithelium into a post-metamorphic sensory system whose epithelial arrangement varies considerably among anurans. In *X. laevis*, for example, the larval main OE lining the principal cavity undergoes extensive cellular remodeling and contributes to the adult air nose, while a new water-sensitive OE develops in the middle cavity (Hansen et al. 1998; Higgs and Burd 2001; Dittrich et al. 2016). In *A. truei*, the PC retains two morphologically distinct epithelial regions, including an anterior larval-type epithelium and a posterior adult-type epithelium, whereas in bufonids and ceratophryids, the water-sensitive epithelium is associated with the recessus olfactorius rather than the middle cavity (Benzekri and Reiss 2012; Jungblut et al. 2017, 2021; Weiss et al. 2021a). *Allobates femoralis* differs from these arrangements in maintaining a continuous main OE along the walls of the PC, without obvious segmentation into separate epithelial regions. The metamorphic remodeling of the main OE can follow several spatial pathways, ranging from regional subdivision or redistribution among olfactory cavities to a continuous epithelial organization within the primary cavity.

In *A. femoralis*, the VNO remains morphologically identifiable throughout larval development and is still evident after metamorphosis, consistent with the occurrence of a distinct VNO across larval and adult stages in other anurans. Its development and organization, however, vary among species (Jungblut et al. 2017, 2021; Weiss et al. 2021a). In *X. laevis*, for example, substantial cell death occurs in the VNO during metamorphosis, despite the continued presence of the organ (Dittrich et al. 2016), indicating that morphological persistence does not necessarily reflect unchanged cellular identity. The VNO as a distinct epithelial structure throughout development in *A. femoralis* particularly relevant for investigating how its cellular organization and sensory properties are maintained or modified during the larval-to-juvenile transition.

### Organization and development of the olfactory bulb

The anuran larval OB is organized into spatially distinct glomerular clusters, including lateral, intermediate, and medial clusters (Nezlin et al. 2003; Manzini et al. 2007; Weiss et al. 2021a). This compartmentalized organization was subsequently examined comparatively in larvae of six anuran species from four families, revealing a corresponding four-cluster arrangement comprising the LC, IC, MC, and DC clusters (Weiss et al. 2020). The recurrence of this four-cluster arrangement across distantly related anurans indicates that the larval OB has a broadly conserved organization, although the relative volume of individual clusters varies among families (Weiss et al. 2020). The OB in larvae of *A. femoralis* similarly consists of four clusters, with the LC, IC, MC, and DC clearly recognizable, further supporting the conservation of this basic larval organization across anuran lineages.

This conserved larval organization is subsequently remodeled during metamorphosis, but the extent and trajectory of these changes differ among anuran species. In *X. laevis*, metamorphosis involves a pronounced reorganization of the olfactory projections associated with changes in the peripheral olfactory epithelia (Weiss et al 2021a). Projections from the larval principal cavity initially terminate in ventral regions of the main OB, whereas projections associated with the newly developing middle-cavity epithelium become incorporated into the ventral OB during metamorphosis. At the same time, olfactory receptor neurons arising in the remodeling principal cavity establish projections to dorsal regions of the OB, within the increasing fusion of the OB hemispheres (Gaudin and Gascuel 2005; Weiss et al. 2021b). Thus, in *X. laevis*, metamorphosis reorganizes both the peripheral sources of olfactory input and their spatial representation within the OB. Comparable developmental observations in *Xenopus tropicalis* and *Dendrobates tinctorius* show that the dorsomedial components converge medially during metamorphosis in both species, whereas the ventral LC, IC, and MC remain identifiable during metamorphosis and subsequently remain relatively stable in *X. tropicalis* but become reduced in *D. tinctorius* (Weiss et al. 2020).

In *A. femoralis*, the LC and DC remain recognizable throughout development, whereas the MC and IC become progressively less distinct during metamorphic stages 42–46. The paired DCs converge medially, forming a prominent fused region that remains evident after metamorphosis. These changes parallel patterns of developmental remodeling described in *X. tropicalis*, *D. tinctorius*, and *X. laevis* (Gaudin and Gascuel 2005; Weiss et al. 2020, 2021b).

Functional evidence, primarily from *X. laevis*, shows that the LC is associated with the lateral olfactory pathway, receiving predominantly microvillous olfactory receptor neuron input and responding to amino acids, suggesting a role in detecting food-related cues (Gliem et al. 2013; Weiss et al. 2021a). A comparable role for lateral olfactory pathways in food-cue detection has been reported in teleosts, where amino acids are prominent food-related odorants (Hamdani et al. 2001; Nikonov et al. 2005; Koide et al. 2009). The persistence of the LC throughout *A. femoralis* development may indicate conservation of this feeding-related olfactory pathway, although anatomical persistence alone cannot confirm the food-related sensory function.

In contrast, the DC, in *X. laevis*, receives input from the main OE and process airborne odorants following the transition to adult life (Weiss et al. 2020, 2021b). Its fusion during late larval development has further been hypothesized to facilitate stereo olfaction and odor-source localization, suggesting a role in the spatial processing of terrestrial odor cues (Weiss et al. 2021b). The prominent fused DC observed in post-metamorphic and juveniles *A. femoralis* may similarly relate to the spatial demands of terrestrial life, including navigation through complex environments and locating water bodies for tadpole deposition (Pašukonis et al. 2016; Serrano-Rojas and Pašukonis 2021; Peignier et al. 2024). Comparable regional specialization of the OB for ecologically relevant behaviors, including attraction and navigation, has also been reported in mammals (Xu et al. 2005; Kang et al. 2009; Gumaste et al. 2024). These comparative patterns provide a functional framework for interpreting the persistent LC and fused DC in *A. femoralis*, but their specific sensory and behavioral significance remains to be tested.

Importantly, interpretation of the metamorphic changes observed in *A. femoralis* should consider that the marker used in the present study is calretinin. The LC and DC were consistently visualized with calretinin throughout development, whereas the reduced visibility of the MC and IC during stages 42–46 cannot by itself establish their anatomical disappearance. Different neuronal markers can label distinct and partially overlapping neuronal populations within the OB and may consequently reveal different aspects of glomerular organization (Crespo et al. 1997; Parrish-Aungst et al. 2007; Moreno et al. 2008). Thus, the present findings establish the developmental pattern of calretinin-positive glomerular population but cannot determine whether the reduced visibility of the MC and IC reflects changes in their neuronal composition, calretinin expression, or underlying organization. Complementary neuronal markers and anatomical tracing will be needed to resolve the basis of these developmental changes.

### Gαo expression

G-protein expression in amphibian olfactory epithelia is closely associated with distinct sensory cell types and therefore provides important information about the organization and functional specialization of olfactory transduction (Weiss et al. 2021a). Gαolf is predominantly expressed by ciliated olfactory receptor neurons (ORNs), an organization particularly well documented in *X. laevis*, where Gαolf-positive ciliated neurons are associated with the adult air nose and the detection of airborne odorants (Mezler et al. 2001; Gliem et al. 2013). In contrast, Gαo is predominantly associated with microvillous sensory neurons and vomeronasal receptor neurons (Hagino-Yamagishi et al. 2004; Gliem et al. 2013; Sansone et al. 2014). This cellular segregation is also evident in *R. arenarum*, where Gαolf- and Gαo-positive neurons form distinct populations within the main OE, whereas the vomeronasal epithelium is strongly dominated by Gαo (Jungblut et al. 2009). Importantly, however, the relationship between G-protein expression, receptor cell morphology, and epithelial identity can be variable. In terrestrial amphibians, Gαolf is also expressed in the vomeronasal epithelium, as demonstrated in *B. japonicus*, where Gαolf- and Gαo-positive neurons occupy different epithelial layers and project to distinct regions of the AOB (Hagino-Yamagishi and Nakazawa 2011), and in *Plethodon shermani*, where Gαolf is predominantly expressed in the main OE but is also detected in the VNO (Kiemnec-Tyburczy et al. 2011). These observations indicate that G-protein expression should not be interpreted solely as a marker of epithelial identity but rather as an indicator of distinct sensory neuron populations and their associated transduction pathways.

A major finding of the present study is the broad distribution of Gαo-expressing receptor neurons across the sensory epithelia of *A. femoralis*. Gαo-positive receptor neurons were detected in the main OE, LA, bOE, and VNO. Importantly, in *A. femoralis*, the detection of Gαo-positive receptor neurons within the LA and bOE provides, to our knowledge, detailed evidence of the molecular identity of the sensory population in these specialized larval epithelia. This finding is particularly relevant because the sensory properties, molecular organization, and transduction mechanisms of both the LA and bOE remain largely unresolved in larval anurans (Jungblut et al. 2021; Weiss et al. 2021a, this study).

Consistent with the broader organization of the amphibian olfactory system, previous studies have shown that Gαo-positive receptor neurons can exhibit distinct projection patterns within the OB among anuran species (Weiss et al. 2021a). In *A. femoralis*, Gαo-positive axons project to both the OB and AOB, and their distribution changes during development in parallel with the remodeling of the olfactory system. In *X. laevis*, Gαo- and Gαi2-positive receptor neurons are associated with glomerular regions of the lateral compartment of the main OB and with the AOB, whereas Gαolf-positive receptor neurons predominantly innervate glomeruli in the intermediate and medial compartments (Gliem et al. 2013). Together, these findings suggest that the Gαo-positive populations identified in *A. femoralis* may comprise multiple functionally distinct receptor neuron populations rather than representing a single anatomically or functionally homogeneous class. However, because the present study characterizes only the Gαo-associated component of the *A. femoralis* olfactory system, the molecular identity of the receptor neuron populations innervating the Gαo-negative regions of the OB remains unresolved, as does their potential association with Gαolf or other G-protein subunits. Characterization of these additional G-protein populations will therefore be important for determining how receptor cell identity, epithelial organization, and central projection patterns are integrated within the olfactory system in *A. femoralis*.

## Conclusion

Overall, our findings show that the olfactory system of *A. femoralis* undergoes extensive developmental remodeling, involving coordinated changes in peripheral sensory epithelia and their central projections during the transition from an aquatic larva to a terrestrial adult. While the overall organization shares several major features with that described in other anurans, the distinct molecular and anatomical features identified here, particularly the differentiation of larval sensory epithelia (i.e., bOE and LA), provide a framework for understanding how these subsystems are organized and potentially specialized. By integrating anatomical and molecular information across development, this study establishes a foundation for functional and behavioral investigations. Specifically, it remains open what olfactory information is detected, how their signals are processed centrally, and how this contributes to the species’ ecology and behavior.

## Supplementary Information

Below is the link to the electronic supplementary material.

(DOCX 1.09 MB)

## Data Availability

All data supporting the findings of this study are available within the paper.
