# Supplementary material for: Organization and remodeling of olfactory subsystems across development in the brilliant-thighed poison frog Allobates femoralis
Source: Cell Tissue Res. 2026 Nov 12;406(1):2. doi: 10.1007/s00441-026-04103-3 (PMC13759571; doi:10.1007/s00441-026-04103-3)
Supplement: Supplementary file 1 [file 441_2026_4103_MOESM1_ESM.docx]

Supporting Information for

**Organization and remodeling of olfactory subsystems across development in the brilliant-thighed poison frog *Allobates femoralis***

Anyelet Valencia-Aguilar¹^*^, [Thomas Hassenklöver](https://onlinelibrary.wiley.com/authored-by/Hassenklöver/Thomas)¹, Eva Ringler², Ivan Manzini¹

¹Department of Animal Physiology and Molecular Biomedicine, Justus-Liebig-Universität Giessen, Heinrich-Buff-Ring 38, 35392 Giessen, Germany

²Division of Behavioural Ecology, Institute of Ecology and Evolution, University of Bern, 3032 Bern, Switzerland

*Corresponding author e-mail: anyelet@gmail.com

**Fig. S1** illustrates the anatomical structure of the olfactory bulb at stage 25 in *Allobates femoralis*. The developing olfactory bulb is divided into clearly separate right and left components. At this early stage, the dorsal olfactory bulb features two distinct clusters, one on each side, with no visible connection across the dorsal midline. The bilateral separation of these dorsal clusters is only observable during pre-metamorphic stages 25–27. Dorsal cluster (DC), medial cluster (MC), intermediate cluster (IC), lateral cluster (LC), and accessory olfactory bulb (AOB). A anterior, P posterior, L left, R right. Immunohistochemical staining for calretinin (green) marks olfactory receptor neurons (ORNs) throughout the olfactory epithelium and their projection to the bulb. Illustration: Javier Lazaro.
